# Multitracer Approach to Understanding the Complexity of Reactive Astrogliosis in Alzheimer’s Brains

**DOI:** 10.1021/acschemneuro.3c00646

**Published:** 2023-12-22

**Authors:** Igor C. Fontana, Amit Kumar, Nobuyuki Okamura, Agneta Nordberg

**Affiliations:** † Division of Clinical Geriatrics, Center for Alzheimer Research, Department of Neurobiology, Care Sciences and Society, Karolinska Institutet, S-141 83 Stockholm, Sweden; ‡ Department of Pharmacology, Tohoku Medical and Pharmaceutical University, Sendai 983-8536, Japan; § Theme Inflammation and Aging, Karolinska University Hospital, S-141 57 Stockholm, Sweden

**Keywords:** Alzheimer’s disease, astrogliosis, PET imaging, deprenyl, SMBT-1, BU99008

## Abstract

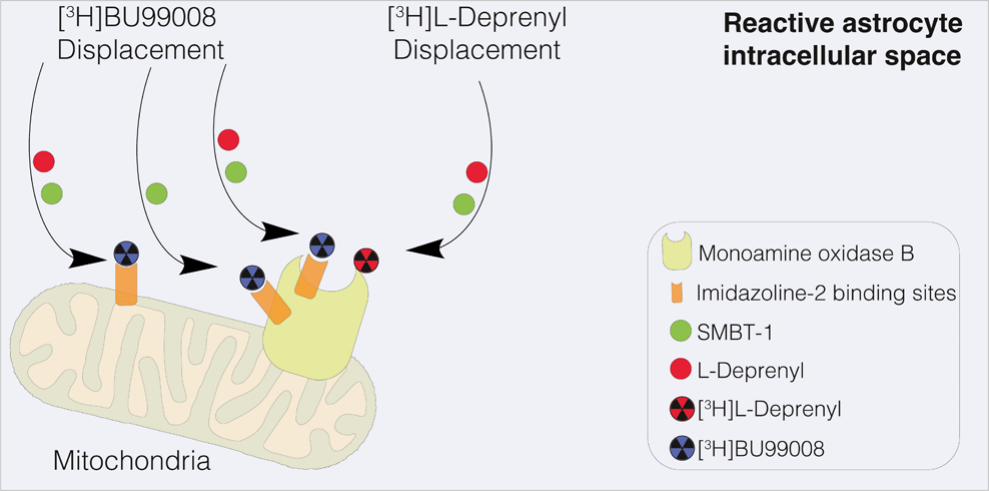

A monoamine oxidase B (MAO-B) selective positron emission tomography (PET) tracer [^11^C]-deuterium-l-deprenyl holds promise for imaging reactive astrogliosis in neurodegenerative diseases, such as Alzheimer′s disease (AD). Two novel PET tracers ([^11^C]-BU99008 and [^18^F]-SMBT-1) have recently been developed to assess the complexity of reactive astrogliosis in the AD continuum. We have investigated the binding properties of SMBT-1, l-deprenyl, and BU99008 in AD and cognitively normal control (CN) brains. Competition binding assays with [^3^H]-l-deprenyl and [^3^H]-BU99008 versus unlabeled SMBT-1 in postmortem AD and CN temporal and frontal cortex brains demonstrated that SMBT-1 interacted with [^3^H]-deprenyl at a single binding site (nM range) and with [^3^H]-BU99008 at multiple binding sites (from nM to μM). Autoradiography studies on large frozen postmortem AD and CN hemisphere brain sections demonstrated that 1 μM SMBT-1 almost completely displaced the [^3^H]-l-deprenyl binding (>90%), while SMBT-1 only partly displaced the [^3^H]-BU99008 binding (50–60% displacement) in cortical regions. In conclusion, SMBT-1, l-deprenyl, and BU99008 interact at the same MAO-B binding site, while BU99008 shows an additional independent binding site in AD and CN brains. The high translational power of our studies in human AD and CN brains suggests that the multitracer approach with SMBT-1, l-deprenyl, and BU99008 could be useful for imaging reactive astrogliosis.

## Introduction

Brain astrocytes are important components in many neurodegenerative diseases. Astrocytic pathological changes represent a multifaceted phenomenon in the brain, which is not limited to reactive astrogliosis, and could range from nonreactive to reactive states in different CNS pathologies (further reading on astrogliopathologies and reactive astrogliosis, see Verkhratsky et al.^1^ and Escartin et al.,^2^ respectively). It might be challenging to measure early reactive astrocytes in brain and, for years, increased glial fibrillary acidic protein (GFAP) immunoreactivity in postmortem tissue has been used as a general marker for reactive astrogliosis.^3^ However, recent evidence in biomarker research indicates that the potential to represent the whole population of reactive astrocytes in the human brain may be lost if testing is confined to changes in GFAP levels.^4,5^ An increase in GFAP levels does not always reflect pathology and can be observed after different physiological stimulation such as enriched environment and physical activity.^1^ In fact, it has been suggested that a heterogeneous population of reactive astrocytes exists, varying among distinct brain regions with specific responses to differing pathologies.^5−7^ Thus, identifying new biomarkers, beyond GFAP, is crucial for capturing the full picture of reactive astrogliosis and improving our understanding of astrocytic heterogeneity in AD.^8^ In this context, overexpression of monoamine oxidase B (MAO-B) has been long put forward as a marker for reactive astrogliosis.^9,10^

These findings encouraged the development of positron emission tomography (PET) tracers, powerful molecular imaging tools that allow specific proteins and metabolic processes to be detected in the living brain in a noninvasive manner, selective for MAO-B, such as [^11^C]-deuterium-l-deprenyl. Indeed, [^11^C]-deuterium-l-deprenyl is a well-established tool for imaging reactive astrocytes in multiple brain diseases, including epilepsy, Creutzfeldt–Jakob disease, and amyotrophic lateral sclerosis.^11−14^ Furthermore, [^11^C]-deuterium-l-deprenyl can detect reactive astrogliosis in the early and late stages of AD.^15−19^

Recently, two novel astrocytic PET tracers have been developed: [^11^C]-BU99008, which targets imidazoline binding site (I_2_B) overexpression in AD brains,^19,20^ and [^18^F]-SMBT-1, which is similar to [^11^C]-deuterium-l-deprenyl with high selectivity for MAO-B. [^11^C]-BU99008 and [^18^F]-SMBT-1 appear to be promising surrogate markers of reactive astrogliosis in AD.^21−25^ However, the binding behavior of SMBT-1, l-deprenyl, and BU99008 has not yet been compared in AD and cognitively normal (CN) brains and requires investigation to improve understanding of the complexity underlying astrocytic heterogeneity and the different subtypes involved in AD. In this work, we used postmortem radioligand assays and brain imaging techniques to further evaluate the potential of SMBT-1 as a novel astrocytic PET tracer in comparison to [^3^H]-l-deprenyl and [^3^H]-BU99008 in CN and AD brains. We hypothesized that SMBT-1 binding mechanisms/behavior should be similar to that of l-deprenyl, displacing only [^3^H]-l-deprenyl in cognitively normal control (CN) and AD brains, with little or no interaction with [^3^H]-BU99008 binding sites.

## Results and Discussion

### [^3^H]-l-deprenyl Competition Binding Assays with Non-radiolabeled SMBT-1 in AD and CN Brains

**Figure 1:**
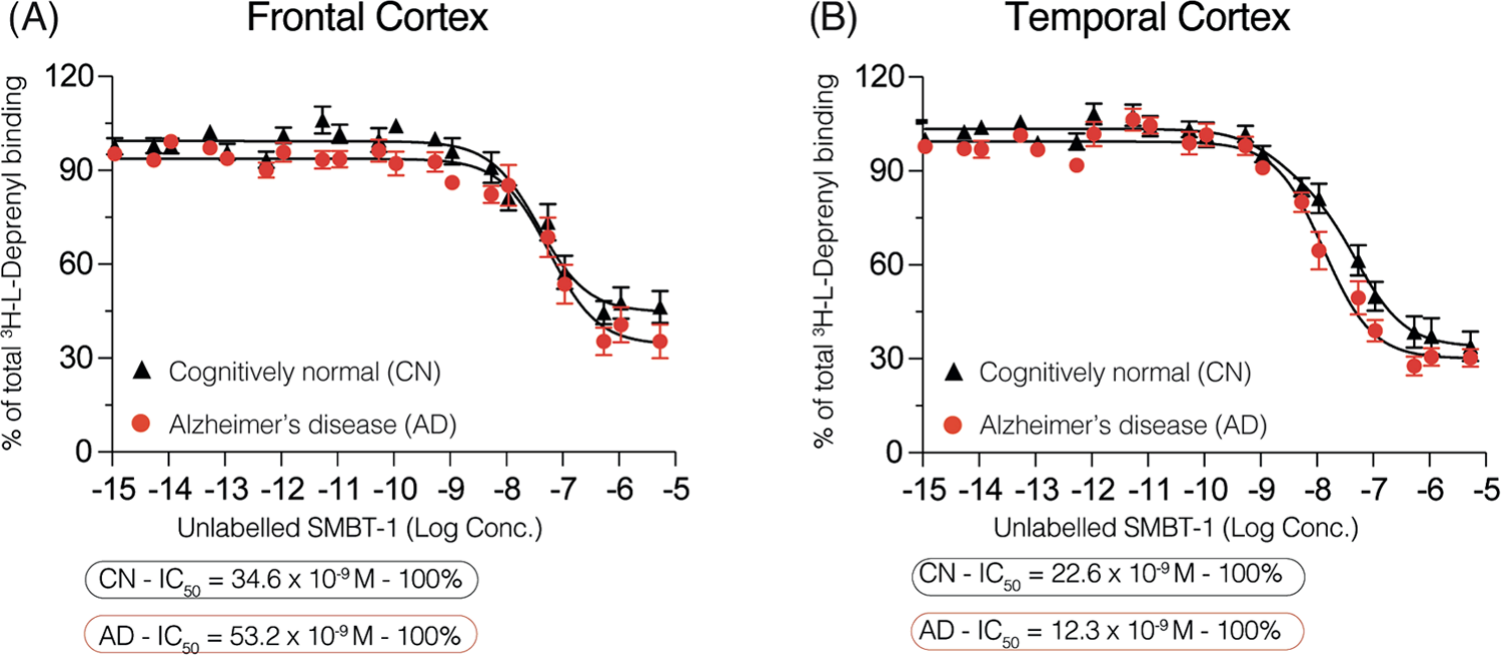
[^3^H]-l-deprenyl competition binding assays with non-radiolabeled SMBT-1 in frontal/temporal cortex CN and AD brain homogenates. Competition binding studies were performed in an increasing concentration range (10^–15^–10^–5^ M) of non-radiolabeled SMBT-1 against a single concentration of [^3^H]-l-deprenyl (10 nM) in (A) frontal and (B) temporal cortex brain homogenates from three CN and three AD subjects. Results are presented as means ± SEM of nine experiments in triplicate. AD, Alzheimer’s disease; CN, cognitively normal control; IC_50_, half-maximal inhibitory concentration. Frontal cortex: *r*^2^ = 0.779 AD and *r*^2^ = 0.768 CN; temporal cortex: *r*^2^ = 0.887 AD and *r*^2^ = 0.857 CN.

We performed competition binding studies in the frontal and temporal cortex brain tissues (3 AD brains and 3 CN brains) to explore and compare the binding behavior of two MAO-B selective PET tracers, SMBT-1 and l-deprenyl. Competition between [^3^H]-l-deprenyl and non-radiolabeled SMBT-1 in the frontal cortex revealed one binding site in the high affinity range for both CN and AD brains (IC_50_ = 34.6 and 53.2 nM, respectively; Figure 1A and Table 1). In the temporal cortex, SMBT-1 showed similar behavior, competing for a single binding site with similar binding affinities in CN (IC_50_ = 22.6 nM) and AD (IC_50_ = 12.3 nM) brains (Figure 1B and Table 1).

**Table 1:** Radioligand Binding Studies in Brain Homogenates^a^

SMBT-1 concentration range versus	group	site 1 (superhigh affinity)	site 2 (high affinity)
[^3^H]-l-deprenyl (10 nM)			
Frontal Cortex			
10^–15^–10^–5^ M	CN	N/A	34.6 × 10^–9^ M
10^–15^–10^–5^ M	AD	N/A	53.2 × 10^–9^ M
Temporal Cortex			
10^–15^–10^–5^ M	CN	N/A	22.6 × 10^–9^ M
10^–15^–10^–5^ M	AD	N/A	12.3 × 10^–9^ M
[^3^H]-BU99008 (1 nM)			
Frontal Cortex			
10^–15^–10^–7^ M	CN	0.20 × 10^–12^ M	9.3 × 10^–9^ M
10^–15^–10^–7^ M	AD	0.20 × 10^–12^ M	187 × 10^–9^ M
Temporal Cortex			
10^–15^–10^–7^ M	CN	0.3 × 10^–12^ M	1015 × 10^–9^ M
10^–15^–10^–7^ M	AD	0.06 × 10^–12^ M	22.7 × 10^–9^ M

a AD, Alzheimer’s disease; CN, cognitively normal control; N/A, not applicable/available.

### [^3^H]-BU99008 Competition Binding Assays with Non-radiolabeled SMBT-1 in AD and CN Brains

**Figure 2:**
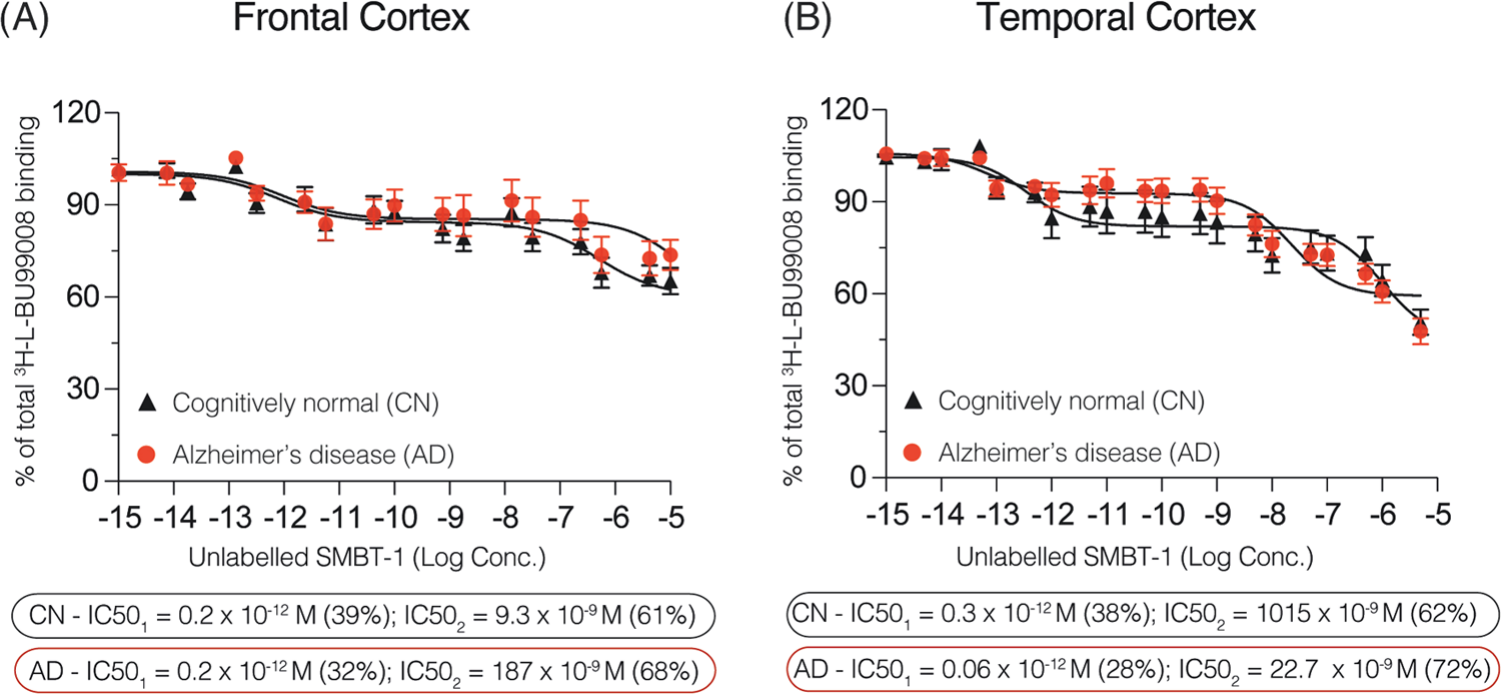
[^3^H]-BU99008 competition binding assays with non-radiolabeled SMBT-1 in frontal/temporal cortex CN and AD brain homogenates. Competition binding studies were performed in an increasing concentration range (10^–15^–10^–5^ M) of non-radiolabeled SMBT-1 against a single concentration of [^3^H]-BU99008 (1 nM) in (A) frontal and (B) temporal cortex brain homogenates from 3 CN and 3 AD subjects. Results are presented as means ± SEM of 9 experiments in triplicate. AD, Alzheimer’s disease; CN, cognitively normal control; IC_50_, half-maximal inhibitory concentration. Frontal cortex *r*^2^ = 0.450 AD and *r*^2^ = 0.553 CN; temporal cortex *r*^2^ = 0.659 AD and *r*^2^ = 0.437 CN.

We performed similar competition binding studies with brain homogenates of the temporal and frontal cortices from 3 AD and 3 CN subjects. In the frontal cortex, the SMBT-1 displacement curve (10^–15^–10^–5^ M) demonstrated two binding sites, as illustrated in Figure 2A: one in the superhigh affinity range (CN IC_50_ = 0.2 pM; AD IC_50_ = 0.2 pM) and one in the high affinity range (CN IC_50_ = 9.3 nM; AD IC_50_ = 187 nM; Table 1). Interestingly, the proportion of superhigh affinity binding sites visualized by SMBT-1 was 68% in the AD frontal cortex and 61% in the CN frontal cortex.

Binding behavior/displacement curves showed differences in the binding affinity of SMBT-1 between the frontal and temporal cortices, especially for AD (Figure 2A,2B and Table 1). Again, analyzing the concentration range of non-radiolabeled SMBT-1 from 10^–15^ to 10^–7^ M, we observed two SMBT-1 binding sites in the temporal cortices of AD and CN brains (Figure 2B). The IC_50_ value for the superhigh affinity binding sites was similar to that of frontal cortex in CN (IC_50_ = 0.3 pM) but lower in AD (and 0.06 pM). Nevertheless, in contrast to our findings in the frontal cortex, the proportion of superhigh affinity binding sites was higher in CN than in AD temporal cortex tissue (62 and 72%, respectively). The second binding site was 1015 nM (i.e., 1 μM) for CN and 22.7 nM for AD.

### SMBT-1 versus [^3^H]-BU99008 and [^3^H]-l-deprenyl (Autoradiography in AD and CN Brains)

**Figure 3:**
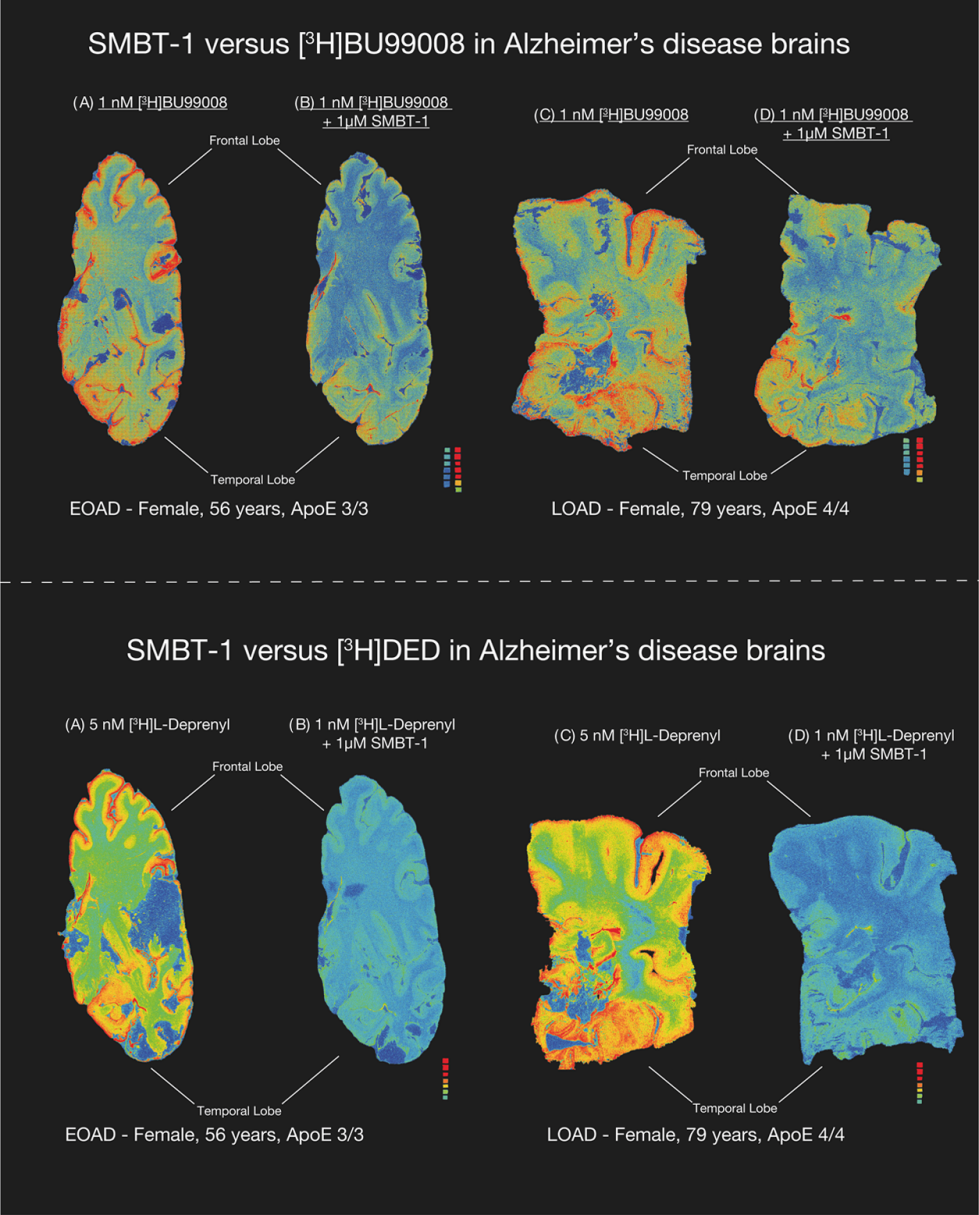
SMBT-1 versus [^3^H]-l-deprenyl and [^3^H]-BU99008 autoradiography on large frozen postmortem AD brain sections. The autoradiograms in the figure show the total binding of 5 nM [^3^H]-l-deprenyl and 1 nM [^3^H] BU99008 in EOAD and LOAD (AD brain coincubation with 1 μM non-radiolabeled SMBT-1 displaced completely [^3^H]-l-deprenyl binding but only partially displaced [^3^H]-BU99008 binding from AD brains). We performed semiquantitative analyses of manually drawn ROI to calculate the total binding values (in fmol/mg) and the % of SMBT-1 displacement. Frontal and temporal lobe regions are marked with dark black bars. Results are presented in Table 2. The autoradiography images were adjusted to standardize the color/threshold levels for comparison. For example, autoradiograms of 5 nM [^3^H]-l-deprenyl and 1 nM [^3^H] BU99008 alone and in the presence 1 μM non-radiolabeled SMBT-1 were at the same level. AD, Alzheimer’s disease; EOAD, early-onset Alzheimer′s disease (<65 years of age); LOAD, late-onset Alzheimer′s disease (>65 years of age).

**Figure 4:**
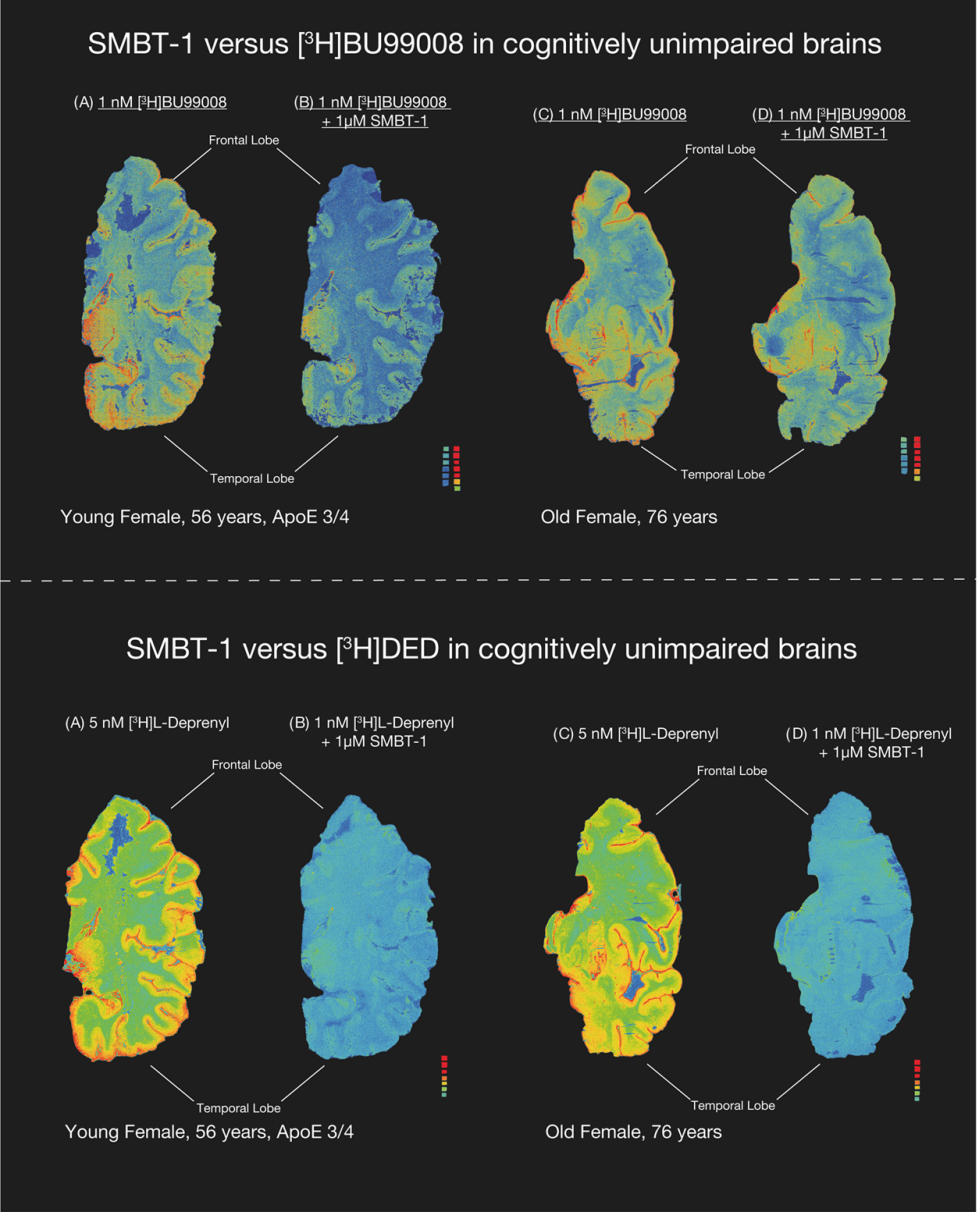
SMBT-1 versus [^3^H] l-deprenyl and [^3^H]-BU99008 autoradiography on large frozen postmortem CN brain sections. The autoradiograms in the figure show the total binding of 5 nM [^3^H]-l-deprenyl and 1 nM [^3^H]-BU99008 in young and old CN brains. Coincubation with 1 μM non-radiolabeled SMBT-1 completely displaced [^3^H]-l-deprenyl binding but only partially displaced [^3^H]-BU99008 binding from CN brains. We performed semiquantitative analyses of manually drawn ROI to calculate the total binding values (in fmol/mg) and the % of SMBT-1 displacement. Frontal and temporal lobe regions are marked with dark black bars. Results are presented in Table 2. The autoradiography images were adjusted to standardize the color/threshold levels for comparison (standards: 0.84–2960 fmol/mg). For example, autoradiograms of 1 nM [^3^H]-BU99008 alone and in the presence 1 μM non-radiolabeled SMBT-1 were at the same level. CN, cognitively normal; ROI, region of interest.

Autoradiograms of large frozen brain sections obtained from one EOAD, one LOAD, and 2 CN subjects demonstrated the regional binding of [^3^H]-BU99008 and [^3^H]-l-deprenyl (total binding) as illustrated in Figures 3 and 4, respectively. The autoradiograms in Figure 3 showed that 1 μM non-radiolabeled SMBT-1 only partly displaced the [^3^H]-BU99008 binding in AD (both EOAD and LOAD) cortical brain regions, while 1 μM non-radiolabeled SMBT-1 completely displaced the [^3^H]-l-deprenyl binding in the AD cortical regions (both EOAD and LOAD), indicating that SMBT-1 and deprenyl seem to compete for the same binding site on MAO-B (Figure 3).

As illustrated in Figure 4 for CN subjects, coincubation of 1 μM SMBT-1 with [^3^H]-BU99008 only partly displaced the [^3^H]-BU99008 binding in the cortical regions, while [^3^H]-l-deprenyl completely displaced the [^3^H]-l-deprenyl binding. The competition data between 1 μM SMBT-1 versus [^3^H]-BU99008 suggest a potential interaction between them, although to a lesser extent, than with [^3^H]-l-deprenyl. Semiquantitative analyses (Table 2) of the young CN large brain sections indicated that SMBT-1 displaced 53% in the temporal cortex and 56% in the frontal cortex. Similarly, in EOAD large brain sections, the displacement was comparable as SMBT-1 displaced 43% of [^3^H]-BU99008 binding in the temporal lobe and 55% in the frontal lobe (compare with Figure 3).

**Table 2:** 1 nM [^3^H]-BU99008 Binding (fmol/mg) in CN and AD Brains Alone (Total) and in the Presence of Unlabeled 1 μM SMBT-1^a^

	young CN			EOAD		
region of interest	total binding	+ SMBT-1	% displaced	total binding	+ SMBT-1	% displaced
temporal lobe	51	24	53	77	44	43
frontal lobe	36	16	56	72	32	55

a CN, cognitively normal control; EOAD, early-onset Alzheimer′s disease (<65 years); young CN < 65 years.

Detecting early brain pathological changes using PET imaging is fundamental to improving AD diagnosis. Over recent decades, a lot of attention has been given to the involvement of glial cells, especially astrocytes, in the early stages of AD. Astrocytes are now considered one of the first brain cells to respond to AD pathology via a phenomenon termed reactive astrogliosis.^15−17^ Following our studies proposing “two waves of reactive astrogliosis” in the AD continuum (see review^26^), immense effort has been directed toward identification and development of novel PET tracers that could improve our understanding of the role of reactive astrocytes in AD pathogenesis. In this context, [^11^C]-deuterium-l-deprenyl and [^11^C]-BU99008, which, respectively, detect overexpression of MAO-B and I_2_Bs in reactive astrocytes, exemplify two available PET radiotracers that image/map reactive astrogliosis in vivo.^16,27^ The recently developed [^18^F]-SMBT-1, which also targets MAO-B with high selectivity, has shown potential as a new surrogate marker for reactive astrogliosis in the AD continuum.^22^ However, a direct evaluation in terms of binding behavior/mechanism between these PET radiotracers in AD and CN brains was still lacking.

Increased MAO-B activity in AD was initially observed and reported four decades ago, in association with brain regions prone to amyloidosis.^9^ The correlation between higher MAO-B levels and reactive astrocytes was later demonstrated by different groups using [^3^H]-l-deprenyl and histological analyses of postmortem brain tissue.^10,28^ Carbon-11 radiolabeling of l-deprenyl allowed reactive astrocytes to be imaged in living individuals.^16,29^ [^11^C]-deuterium-l-deprenyl PET demonstrated higher binding in patients with mild cognitive impairment or presymptomatic autosomal-dominant AD (compared to CN individuals), suggesting that reactive astrogliosis is an early phenomenon in the AD continuum.^15−17^ An increased MAO-B activity has also been demonstrated by [^11^C]-deuterium-l-deprenyl PET in the physiological aging of normal healthy individuals (35). In a recent cross-sectional study, high [^18^F]-SMBT-1 binding has been demonstrated in regions linked with early amyloid-β (Aβ) deposition in AD patients and Aβ+ CN subjects.^22^ To examine SMBT-1 binding behavior and its interaction with [^3^H]-l-deprenyl binding sites, we undertook competition binding assays using a very broad concentration range of non-radiolabeled SMBT-1 (10^–15^–10^–5^ M) against [^3^H]-l-deprenyl in brain homogenates from the temporal and frontal cortices of AD and CN brains. We observed very similar displacement curve patterns, with one binding site (in the nanomolar range) in both analyzed brain regions of AD and CN brains. These findings correlated very well with our previous competition studies in temporal cortex brain homogenates with [^3^H]-l-deprenyl versus non-radiolabeled l-deprenyl, where we also observed one binding site (with nanomolar affinity) in both AD and CN brains.^19^ Furthermore, in this work, we have demonstrated, using large frozen hemisphere section autoradiography studies of AD postmortem brain tissue, that [^3^H]-l-deprenyl binding is completely displaced/abolished by micromolar concentrations of SMBT-1 in the cortical regions. These findings clearly suggest that l-deprenyl and SMBT-1 selectively compete for the same site (i.e., MAO-B) on reactive astrocytes. Our results are in line with those of Harada et al., who used small brain section autoradiography to show that [^18^F]-SMBT-1 binding was fully displaced by the non-radiolabeled selective MAO-B inhibitor lazabemide.^24^

The I_2_B-selective PET tracer [^11^C]-BU99008 is another potential tool for exploring different astrocytic states and reactive astrogliosis. Increased I_2_B density, which is often observed in astrocytes, is associated with GFAP upregulation and reactive astrogliosis in AD.^20^ We have previously demonstrated that [^3^H]-BU99008 can visualize reactive astrogliosis and detect multiple binding sites in AD brains.^19^ We have also reported that the binding behavior of [^3^H]-l-deprenyl is different from that of [^3^H]-BU99008 and that they might be targeting distinct astrocytic subpopulations or states. Consequently, we decided to investigate the binding behavior of SMBT-1 in the context of [^3^H]-BU99008, as we have previously done for l-deprenyl versus [^3^H]-BU99008.^19^ Competition radioligand assays in brain homogenates revealed that SMBT-1 could also interact with [^3^H]-BU99008 at two binding sites with varying affinities in the frontal and temporal cortices of both the AD and CN brains. The binding affinities ranged from picomolar to nanomolar, and the total binding displacement was ∼50%. These results corroborate our previous findings, where l-deprenyl also interacted with [^3^H]-BU99008 at multiple binding sites with pico- and micromolar affinities.^19^ The autoradiograms of large sections of AD brain tissue provided complementary data into the interaction between SMBT-1 and [^3^H]-BU99008 binding sites and indicated displacement of [^3^H]-BU99008 binding in both frontal and temporal lobes in a similar extent to that observed in the brain homogenate studies (43–56%). Remarkably, the proportional displacement of [^3^H]-BU99008 by SMBT-1 in competition studies differed between the frontal and temporal cortices. These changes in the proportion of binding could be attributed to the existence of I_2_Bs in the catalytic site of MAO-B^30^ and, to some extent, to conformational changes in the protein structure, perhaps pathologically induced and in specific regions of the brain. A detailed crystallography analysis led by Bonivento and colleagues^31^ showed that tranylcypromine (an irreversible MAO-B inhibitor) induces conformational changes in the enzyme, leading to the formation of a high-affinity I_2_B.^31^ In this context, it is possible that the nanomolar high affinity site detected by SMBT-1 could be the I_2_B for [^3^H]-BU99008 binding in reactive astrocytes.^19^ Similarly, we also observed the existence of a binding site in the picomolar range (superhigh affinity). The interaction at the picomolar site agrees with our previous findings and conclusions: the existence of an MAO-B site to which BU99008 can bind and be blocked with MAO inhibitors.^19^ In our competition studies using non-radiolabeled SMBT-1 concentrations in the range of 10^–12^–10^–5^ M with [^3^H]-BU99008, we observed, in addition to a superhigh and high affinity site, a third low affinity site in the micromolar range in both the analyzed regions. This low affinity site most probably could represent a nonspecific binding of SMBT-1 binding to additional non-MAO binding sites for BU99008 and, hence, of no interest in PET studies.

These results show that subtle alterations in the biological milieu can affect the MAO-B conformation and, consequently, the number of available sites and the ligand binding affinity. In addition, the regional differences observed suggest that dynamic changes in the [^3^H]-BU99008 binding sites in the pico- and nanomolar affinity ranges could also be dependent on the brain region. However, these observations/conclusions need further exploration. If we look from a broader perspective, these differences in tracer binding resulting from different astrocytic subtypes or states could be a benefit in disguise as a multi-PET approach with different astrocytic tracers could be employed to deepen our understanding of astrocytic heterogeneity in the AD continuum. The changes in MAO-B activity measured by different PET tracers in vivo in AD might represent defense mechanisms aimed at modulating neuronal excitability by increasing tonic glial GABA inhibition.^32^

Our studies have some limitations: first, the binding studies were performed on a small number of cases and a follow-up study in a large cohort of AD cases would be interesting to further validate the potential and binding characteristics of SMBT-1 in relation to l-deprenyl and BU99008. However, despite this limitation, the findings presented here clearly justify the aims of this explorative study. Second, caution should be exercised when directly comparing the results of different autoradiography studies as the large frozen brain sections could be from different coronal anatomical levels and may have scarring in some regions. Overall, our findings have shown that SMBT-1 behaves like l-deprenyl and that it could target MAO-B with high selectivity in AD brains. Moreover, SMBT-1 could also interact with BU99008 at multiple binding sites, possibly with different outcomes in AD and CN brains.

**Table 3:** Clinical Demographic Information for the Brain Donors^a^

group	sex (M/F)	age (years)	braak stage	ApoE (E/E)	onset	postmortem delay (h:min)
For Brain Homogenate Binding Studies						
CN	F	50	1	3/3	N/A	4:10
CN	F	77	1	3/3	N/A	2:55
CN	M	79	2	3/3	N/A	9:00
AD	F	59	5	4/4	EOAD	4:20
AD	M	78	5	4/4	LOAD	6:35
AD	F	85	4	3/3	LOAD	6:00
For Autoradiography Binding Studies						
CN^b^	F	56	N/A	3/4	N/A	2:56
CN^b^	F	76	1	N/A	N/A	4:00
AD^b^	F	57	N/A	3/3	EOAD	2:48
AD^b^	F	79	5	4/4	LOAD	16:00

a AD, Alzheimer’s disease; ApoE, apolipoprotein E; CN, cognitively normal control; EOAD, early onset Alzheimer’s disease, F, female, LOAD, late-onset Alzheimer’s disease; M,male; N/A, not applicable/available.

b Clinical and neuropathological data have been described in earlier publications.^19,34,39,40^

## Conclusions

Identifying early biological changes in the brain of AD patients is an urgent need to foster the development of novel, disease-modifying therapies. In this context, targeting reactive astrocytes is a strategy that goes beyond the classical view of protein misfolding as a single entity in AD pathogenesis. Nevertheless, before developing therapeutic tools to modulate astrocyte function in AD, we need to fully understand how these cells react in the different stages of the AD continuum and for that, new astrocytic PET tracers are required.^33^ In summary, our study highlights the complexity of targeting reactive astrogliosis in AD brains and indicates that a multi-PET approach using the different astrocytic PET tracers presented here could be a way forward in understanding the role of reactive astrogliosis and in targeting astrocytic heterogeneity or subtypes in the AD continuum.

## Methods

### Chemicals

[^3^H]-BU99008 [specific activity (SA) = 3034 MBq/μmol] and [^3^H]-l-deprenyl (SA = 3034 MBq/μmol) were synthesized by Novandi Chemistry AB (Södertälje, Sweden). Non-radiolabeled SMBT-1 was synthesized in-house at Tohoku University as described previously.^21^ Other chemicals [sodium chloride (NaCl), potassium chloride (KCl), calcium chloride (CaCl_2_), Tris base, magnesium chloride (MgCl_2_), disodium phosphate (Na_2_HPO_4_) and potassium dihydrogen phosphate (KH_2_PO_4_)] were acquired from Sigma-Aldrich AB, Sweden.

### Human Postmortem Brain Tissue

Frozen human brain tissue from AD patients and CN subjects was acquired from The Netherlands Brain Bank, Amsterdam, The Netherlands (see Table 3 for clinical information). All brains were obtained with only short postmortem delay and kept frozen at −80 °C until use. Temporal and frontal cortex brain homogenates were prepared in 1× phosphate-buffered saline (PBS) buffer (pH 7.4) with 0.1% bovine serum albumin (BSA) and protease/phosphatase inhibitors and were stored at −80 °C in aliquots until used in the competition binding assays. For the autoradiography experiments, large frozen brain tissue specimens comprising the whole right hemisphere of one early-onset AD (EOAD) and two CN (please refer to Table 3 for clinical information) were provided by the Neuropathology of Dementia Laboratory, Indiana University School of Medicine, Indianapolis, IN. One right hemisphere from a single late-onset AD (LOAD) was provided by the Brain Bank at Karolinska Institutet, Sweden.

### Competition Binding Experiments

Competition binding assays for [^3^H]-l-deprenyl or [^3^H]-BU99008 versus SMBT-1 were performed in postmortem temporal and frontal cortex brain homogenates from 3 AD cases and 3 CN subjects as previously described.^19,34,35^ In brief, 0.1 mg of brain homogenate solution was incubated with a single concentration of [^3^H]-l-deprenyl (10 nM) or [^3^H]-BU99008 (1 nM) and increasing concentrations of non-radiolabeled SMBT-1 (10^–15^–10^–5^) in each specific buffer (for [^3^H]-BU99008, 50 mM Tris-HCl buffer, pH 7.4; for [^3^H]-l-deprenyl, 50 mM Na–K phosphate buffer, pH 7.4) for 1 h at 37 °C in a water bath. After incubation, the reaction was stopped by filtering the mixture through glass fiber filters (previously soaked in 0.3% polyethylenimine solution for 3 h), followed by three quick rinses with cold buffer and overnight incubation of the filter paper at RT in scintillation liquid. Next day, the radioactivity in the filters was counted using a β scintillation counter (PerkinElmer Tri-Carb 2910TR). The data were analyzed using the nonlinear regression function of GraphPad Prism Software version 9.3.1 (350) for Mac OSX to determine the number of binding sites and their affinities (IC_50_) as well as the proportions in AD and CN brains.

### In Vitro Autoradiography Competition Binding Studies

Autoradiography studies were carried out on large frozen postmortem brain sections from the following cases: one EOAD, one LOAD, and CN (one <65 years and one >65 years), as previously described.^19,34,36^ To obtain the large brain slices, frozen sections (100 μm in thickness) were cut from tissue blocks using a Leica CM 3600 XP cryostat and placed on SuperFrost glass slides (SuperFrostPlus, MenzelGläser, Germany).^37,38^ The slides were kept frozen at −80 °C until use. For each experiment ([^3^H]-BU99008 or [^3^H]-l-deprenyl versus non-radiolabeled SMBT-1), two large frozen sections were allowed to dry at room temperature (RT) for 45–60 min, followed by 1 h incubation with either [^3^H]-BU99008 (1 nM) or [^3^H]-l-deprenyl (5 nM) at RT (for total binding: [^3^H]-radiolabeled only and for displacement binding analyses: [^3^H]-radiolabeled + 1 μM non-radiolabeled SMBT-1). To remove excess radiolabeled compounds, the sections were gently rinsed three times (5 min) with the cold specific buffer (for [^3^H]-BU99008, 50 mM Tris-HCl buffer, pH 7.4; for [^3^H]-l-deprenyl, 50 mM Na–K phosphate buffer, pH 7.4). After washing, the sections were quickly dipped into cold Mili Q water and allowed to dry for 24 h at RT. The dried sections were exposed with a tritium standard (Larodan Fine Chemicals AB, Mälmo, Sweden) on a phosphor plate for 4 days for [^3^H]-l-deprenyl and 7 days for [^3^H]-BU99008. After the indicated exposure time, the sections were imaged by using a BAS-2500 phosphor imager (Fujifilm, Tokyo, Japan). A software multigauge was used to manually draw the regions of interest (ROIs) on the autoradiogram for semiquantitative analysis. Using the standard curve, photostimulated luminescence per square millimeter (PSL/mm^2^) was converted into fmol/mg to determine the total and displacement binding (in the presence of 1 μM non-radiolabeled SMBT-1) of [^3^H]-l-deprenyl and [^3^H]-BU99008 in each ROI (Table 3).

## Data Availability

All data generated or analyzed during this study are included in this published article.

## Notes

The study was conducted according to the principles of the Declaration of Helsinki and subsequent revisions. All experiments on autopsied human brain tissue were carried out in accordance with ethical permission obtained from the regional human ethics committee in Stockholm (Permission Numbers 2011/962/31-1, 2006/901-31/3, and 2017/2301-32), and the medical ethics committee of the VU Medical Center for The Netherlands Brain Bank tissue (Permission Number 1998-06/5), Indiana University Institutional Review Board, USA.

## Notes

Previous consent to publish the results of experiments was given at the time of brain donation, and no supplementary consent was needed for this study.

## Abbreviations

AD: Alzheimer’s disease
Aβ: amyloid-β
BSA: bovine serum albumin
CN: cognitively normal
GFAP: glial fibrillary acidic protein
IC_50_: half-maximal inhibitory concentration
I_2_B: imidazoline binding site
MAO-B: monoamine oxidase B
PBS: phosphate buffer saline
PSL: photostimulated luminescence
PET: positron emission tomography
ROI: region of interest
RT: room temperature
SA: specific activity
